
JOURNAL INFORMATION
==============================
NLM Title Abbreviation: ScientificWorldJournal
Journal ID: ScientificWorldJournal
NLM Journal ID: 101131163
Journal ID: TSWJ

The Scientific World Journal

ISSN: 2356-6140
EISSN: 1537-744X
Publisher: Wiley

ARTICLE INFORMATION
==============================
PMCID: PMC6009418
PMID: 29973833
DOI: 10.1100/tsw.2002.58
Article ID: 623968
Article version: 1
Subjects: Short Report

Hypoxia-Inducible Factor-1 (Hif-1) in Experimental Brain Ischemia

Schmidt-Kastner Rainald 1 *
Zhang Bao-Tong 1
Webster Keith 2
Kietzmann Thomas 3
Zhao Weizhao 1
Busto Raul 1
Ginsberg Myron D. 1
Deptartments. of Neurology, University of Miami School of Medicine, P.O. Box 016960, Miami, FL 33101, USA
Mol. Cell. Pharmacology, University of Miami School of Medicine, P.O. Box 016960, Miami, FL 33101, USA
Inst. Biochem. Mol. Cell Biol., Univ. Göttingen, Germany
*Rainald Schmidt-Kastner: rainald@stroke.med.miami.edu
Publication date: 2002
Electronic publication date: 2002 Apr 9
Volume: 2
First page: 123
Last page: 124
Copyright: Copyright © 2002 Rainald Schmidt-Kastner et al.
Copyright year: 2002
License: This is an open access article distributed under the Creative Commons Attribution License, which permits unrestricted use, distribution, and reproduction in any medium, provided the original work is properly cited.
License URL: https://creativecommons.org/licenses/by/3.0/

Funding: National Institutes of Health NS05820

==============================
Advances in Gene Technology: The Genome and Beyond –
Structural Biology for Medicine (Proceedings of the 2002
Miami *Nature Biotechnology* Winter Symposium)
*TheScientificWorld* 2002, 2(S2), 123–124
ISSN 1532-2246; DOI 10.1100/tsw.2002.58

### HYPOXIA-INDUCIBLE FACTOR-1 (HIF-1) IN EXPERIMENTAL BRAIN ISCHEMIA

Rainald Schmidt-Kastner[*,1], Bao-Tong Zhang[1], Keith Webster[2], Thomas Kietzmann[3], Weizhao Zhao[1], Raul Busto[1], and Myron D. Ginsberg[1]
[1]Deptartments. of Neurology and [2]Mol. Cell. Pharmacology, University of Miami School of Medicine, P.O. Box 016960, Miami, FL 33101; [3]Inst. Biochem. Mol. Cell Biol., Univ. Göttingen, Germany
*Rainald@stroke.med.miami.edu

**INTRODUCTION.** Recently, a specific defense system against hypoxia has been described which uses the hypoxia-inducible factor-1 (HIF-1) as transcription factor. HIF-1 coordinates the response to prolonged hypoxia which pertains to glycolysis (e.g., lactate dehydrogenase = LDH-5), glucose transport, vasodilation, and angiogenesis[1]. The level of the HIF-1α subunit is oxygen-dependent, and the protein concentration is mainly regulated via degradation in the proteasome[1], involving a novel class of oxygen-sensing proline-4-hydroxylases[2]. HIF-1α combines with the constitutively expressed HIF-1β protein to form HIF-1[1]. Ischemia and hypoxia of the brain are major events in cardiac arrest and stroke. In this report, we examined changes of HIF-1α mRNA and the target gene, LDH-5, in the forebrain of rats after transient global brain ischemia[3] or in chronic oligemia[4].

**METHOD**. Global brain ischemia was induced in halothane-anesthetized, normothermic Wistar rats by using two-vessel occlusion and hypotension for 12.5 min. The recirculation periods were 30 min, 1, 3, 6 h, and 1, 2, 3, and 7 days (n = 2-5). To induce chronic oligemia, permanent bilateral common carotid artery occlusion (BCCAO) was used in Wistar rats for 1 h, 6 h, 1 day, and 7 days (n = 4-5). Rat-specific polyclonal antibodies for HIF-1α were used in immunohistochemistry[5] on vibratome sections of 4% paraformaldehyde-fixed brains. *In situ* hybridization was carried out using radioactive, rat-specific antisense probes to HIF-1α mRNA and LDH-5 mRNA[6], followed by quantitative film autoradiography.

**RESULTS.** Using a rat-HIF-1α specific polyclonal antiserum in immunohistochemical studies on control rat brains, we obtained generalized neuronal labelling in forebrain regions; astrocytes or vessels were unreactive. In parallel, *in situ* hybridization showed widespread expression of HIF-1α mRNA in control brains. HIF-1α mRNA was increased in CA1 at 1 day after global ischemia (optical density = 197 ± 14 % of control; ANOVA, $p < 0.05$) but not at earlier time points. The increased signal was clearly localized to the vulnerable CA1 neuronal layer in emulsion-coated sections. HIF-1α-like immunoreactivity slightly decreased in CA1 by 1 day, and then disappeared at 3 and 7

days due to cell death. No increase in HIF-1α mRNA or LDH-5 mRNA was found in regions with reactive glial changes. By contrast, a rapid and widespread increase of HSP70 mRNA was noted in the forebrain between 30 min and 1 day after global brain ischemia. In the oligemia model, constant levels of HIF-1α mRNA were observed, whereas LDH-5 mRNA was upregulated in the neocortex at 1 day with a columnar pattern.

**DISCUSSION.** Our study indicates that HIF-1α is constitutively expressed in forebrain neurons, implying that neurons can perform immediate regulatory responses to hypoxia and ischemia. The presently used HIF-1α antibodies indicate a high expression level in neurons, while detection of active or degraded forms of HIF-1α remains to be defined. Upregulation of HIF-1α mRNA occurred at 1 day of recirculation, and it was limited to the vulnerable CA1 neurons that are bound to die by 2–3 days after ischemia. Thus, HIF-1 regulation differs from a typical stress response gene such as HSP70 that is expressed early after ischemia and in a widespread fashion. It remains to be determined whether an additional hypoxic situation preceded cell death in CA1 neurons and whether changes in HIF-1α mRNA are in reaction to a consumption of HIF-1 protein. Oligemia per se affected the HIF-1 target gene, LDH-5, suggesting that hypoxia prevailed in the chronic carotid occlusion model.

**ACKNOWLEDGEMENT.** Supported by NS05820.

**REFERENCES**

1. Semenza, G.L. (1998) Curr. Opin. Genet. Dev. 8, 588–594.
2. Epstein, A.C.R. et al. (2001) Cell 107, 43–54.
3. Schmidt-Kastner, R., Fliss, H., and Hakim, A.M. (1997) Stroke 28, 163–170.
4. Schmidt-Kastner, R., Truettner, J., Lin, B., Zhao, W., Saul, I., Busto, R., and Ginsberg, M.D. (2001) Mol. Brain Res. 92, 157–166.
5. Kietzmann, T., Roth, U., and Jungermann, K. (1999) Blood 94, 4177–4185.
6. Webster, K. (1987) Mol. Cell. Biochem. 77, 19–28.
